# The Effect of 42-Day Exposure to a Low Deoxynivalenol Dose on the Immunohistochemical Expression of Intestinal ERs and the Activation of *CYP1A1* and *GSTP1* Genes in the Large Intestine of Pre-pubertal Gilts

**DOI:** 10.3389/fvets.2021.644549

**Published:** 2021-07-19

**Authors:** Magdalena Gajęcka, Paweł Brzuzan, Iwona Otrocka-Domagała, Łukasz Zielonka, Sylwia Lisieska-Żołnierczyk, Maciej T. Gajęcki

**Affiliations:** ^1^Department of Veterinary Prevention and Feed Hygiene, Faculty of Veterinary Medicine, University of Warmia and Mazury in Olsztyn, Olsztyn, Poland; ^2^Department of Environmental Biotechnology, Faculty of Environmental Sciences and Fisheries, University of Warmia and Mazury in Olsztyn, Olsztyn, Poland; ^3^Department of Pathological Anatomy, Faculty of Veterinary Medicine, University of Warmia and Mazury in Olsztyn, Olsztyn, Poland; ^4^Independent Public Health Care Center of the Ministry of the Interior and Administration and the Warmia and Mazury Oncology Center in Olsztyn, Olsztyn, Poland

**Keywords:** deoxynivalenol, intestines, ERα and ERβ immunohistochemistry, colon, activation of *CYP1A1* and *GSTP1* genes

## Abstract

Deoxynivalenol (DON) is a mycotoxin that contaminates various plant materials. Exposure to DON can disrupt hormonal homeostasis, decrease body weight gains and modulate the immune system in pigs. It can also cause diarrhea, vomiting, leukocytosis, hemorrhaging or even death. Prolonged exposure to low doses of DON can have serious health implications in mammals. This is the first *in vivo* study to show that *per os* administration of low DON doses probably contributes to specific dysfunctions in steroidogenesis processes by inducing the immunohistochemical expression of estrogen receptors alpha (ERα) in the entire gastrointestinal tract in strongly stained cells (3 points) and estrogen receptors beta (ERβ), but only in both investigated segments of the duodenum in pre-pubertal gilts. Therefore, the aim of this study was to determine whether a NOAEL dose of DON (12 μg DON/kg BW) administered *per os* over a period of 42 days induces changes in the immunohistochemical expression of ER in different intestinal segments and the transcriptional activation of *CYP1A1* and *GSTP1* genes in the large intestine of pre-pubertal gilts. This is the first report to demonstrate the expression of ER, in particular ERβ, with the associated consequences. The expression of ER was accompanied by considerable variations in the activation of *CYP1A1* and *GSTP1* genes, but it supported the maintenance of a stable consensus between the degree of mycotoxin exposure and the detoxifying effect in pre-pubertal gilts.

## Introduction

Mycotoxins are substances that occur naturally in the environment (1, 2). In addition to mycoestrogens such as zearalenone (3, 4), selected trichothecenes, including DON and/or its metabolites (but *in vitro*), can affect steroidogenesis (2) and changes in gene expression, which suggests that these compounds could disrupt hormonal homeostasis (1).

Deoxynivalenol (DON), a polar organic compound produced mainly by *Fusarium graminearum* and *Fusarium culmorum* (5), is one of the most economically important mycotoxins in the world (6). The presence of a ketone group at position C8 is characteristic of B trichothecenes. Deoxynivalenol also contains an epoxide group which enables the mycotoxin to bind to a large number of eukaryotic ribosome subunits and disrupt the activity of peptide transferase, thus compromising the elongation or shortening of peptide chains (7). Deoxynivalenol affects the cellular transport rate and enzyme metabolism in the cytoplasm (8), it leads to changes in affinity for active binding sites and disrupts protein synthesis (2).

There is a general scarcity of *in vitro* and, in particular, *in vivo* evidence that DON can directly trigger the overexpression of steroid hormone receptors or induce hormonal activity. However, DON affects steroidogenesis (2) and changes in gene expression (9), which suggests that it can potentially disrupt the functioning of the hormonal system. The available background knowledge indicates that estrogen receptors (ER) are essential for various physiological processes, including the regulation of the endocrine system, lipid profile, bone integrity, hemostasis and endothelial function. Estrogen receptors are inflammatory markers, and they promote the growth of various tissues, and contribute to pre-natal and post-natal development (10). Estrogen receptors are divided into two subtypes, ERα and ERβ, which have different identity (11) and interact with different tissues. For example, ERα is more prevalent in the gonads, mammary glands, kidneys and bronchi, and it stimulates proliferative processes in cells. In turn, ERβ is predominant in bones and pulmonary alveoli, and it delivers antiproliferative effects (12, 13). Unlike other steroid receptors, ERs bind to a wide range of exogeneous compounds, and are they are increasingly often classified as promiscuous receptors (14). Ligand recognition and receptor-ligand binding are regarded as highly specific biological processes that activate the appropriate receptors and trigger specific responses to stimuli *in vivo*. It is believed that protein-ligand recognition relies on a lock-and-key model and an improved induced-fit model. Estrogen receptors can bind to various ligands due to their unique ability to associate ligands and detect binding cavities. They appear to be an exception to the core concept of lock and key, and they do not discriminate between the bound ligands. It remains unknown whether the above mechanism also applies to non-steroid compounds. Estrogen receptors have evolved to play different tissue-dependent biological roles (15).

The fact that DON is not involved in phase I biotransformation reactions (16), and P450 isoenzymes, in particular CYP1A1, participate in the metabolism of many structurally different endogenous and exogenous compounds, especially the hydroxylation of estrogens (17), in distal segments of the intestines, had to be considered in the present research. Nandekar et al. (18) suggested that the *CYP1A1* gene in macroorganisms undergoes transcriptional activation under exposure to environmental contaminants (such as DON), in particular substrates of the CYP1A1 enzyme. Recent *in vitro* studies have demonstrated that CYP1A1 can also act as a detoxifying enzyme (9).

Glutathione S-transferase (GST), also known as ligandin, is a phase II biotransformation enzyme and a biomarker of plant contamination (19) with undesirable substances such as mycotoxins (9). Glutathione S-transferase isoenzymes are generally overexpressed under exposure to substances which modulate apoptotic processes and induce the expression of biologically active proteins, as well as during detoxification processes (20).

The above problems were considered in the process of formulating the research objective. This study set out to determine whether DON can influence the expression of ER and genes encoding selected large intestine enzymes which participate in the detoxification of mycotoxins. A pioneering *in vivo* study was conducted to verify the research hypothesis. The presented research is innovative because it aimed to determine whether the presence of a NOAEL dose of DON, which is not a contributing factor to clinical mycotoxicosis, can induce subclinical states such as steroidogenesis dysfunction or physiological detoxification. The aim of this study was to determine *in vivo* whether a low dose of DON administered *per os* for 42 days can affect the immunohistochemical expression of ER in different intestinal segments on different dates of exposure or the expression of genes (*CYP1A1* and *GSTP1*) encoding selected enzymes in the colon of pre-pubertal gilts.

## Materials and Methods

### Experimental Animals

The experiment, conducted at the Department of Veterinary Prevention and Feed Hygiene, Faculty of Veterinary Medicine (University of Warmia and Mazury in Olsztyn, Poland), involved 36 pre-pubertal gilts. The animals were clinically healthy, and had initial BW of 25 ± 2 kg. They were kept in group pens, with free access to water. The feed offered to gilts had been tested for the presence of the following mycotoxins: DON, ZEN and α-ZEL. Mycotoxin concentrations were determined by separation techniques, using immunoaffinity columns (DON-Test^TM^ DON Testing System, VICAM, Watertown, USA; Zearala-Test^TM^ Zearalenone Testing System, G1012, VICAM, Watertown, USA) and HPLC (Hewlett Packard, type 1,050 and 1,100) (21) with fluorescence and/or UV detection [limit of detection: 1.0 ng/g for DON–(22); 3.0 ng/g for ZEN–(23)].

### Experimental Design

The animals were divided into an exposed group (E = DON; *n* = 18) and a control group (C, *n* = 18) (24). Group E gilts were administered DON *per os* at 12 μg/kg BW – this value was consistent with the limits set by EFSA (25) and the NOAEL dose when the experiment was planned. Group C animals received a placebo. Deoxynivalenol was administered daily, before morning feeding, in gastro-soluble gel capsules, with feed as the carrier. The animals in group C received the same gel capsules, but without the mycotoxin.

The analyzed mycotoxin (DON) was biosynthesized, purified and standardized at the Department of Chemistry, Poznań University of Life Sciences (Poland). The experiment lasted for 42 days. The DON dose was adjusted to the BW of pre-pubertal gilts. In order to prevent uneven feed intake, DON was administered in capsules Deoxynivalenol samples were diluted in 500 μL of 96% ethyl alcohol (SWW 2442-90, Polskie Odczynniki Chemiczne SA, Poland) to achieve the required dose (based on the BW of animals). Final solutions were stored for 12 h at room temperature to evaporate the solvent. During the adjustment period, the animals were weighed at 7-day intervals. Three gilts from the exposed and control groups (a total of six animals) were euthanized by intravenous administration of pentobarbital sodium (Fatro, Ozzano Emilia BO, Italy) and bleeding on experimental days 7 (week I), 14 (week II), 21 (week III), 28 (week IV), 35 (week V) and 42 (week VI). Immediately after cardiac arrest, sections of intestinal tissues were collected for analyses.

### Reagents

#### Deoxynivalenol

Deoxynivalenol was biosynthesized at the Department of Chemistry, Poznań University of Life Sciences (Poland), in accordance with a previously described procedure (26, 27).

#### Chemicals and Apparatus

A chromatographic analysis of DON was conducted at the Department of Chemistry, Poznań University of Life Sciences (Poland), in accordance with a previously described procedure (22).

### Immunohistochemistry

#### Tissue Samples

Every week, post-mortem samples of porcine gastrointestinal tissues were rinsed with phosphate buffer. Cross-sectional tissue samples (measuring ~1 × 1.5 cm, two samples from each examined tissue) were obtained from: the duodenum – the first part (duodenal cap) and the horizontal or third part; jejunum and ileum – middle parts; cecum – 1 cm from the ileocecal valve; and the colon – middle parts of the ascending colon and descending colon.

#### Location of ERα and ERβ

The variability in ER expression was assessed in intestinal epithelial cells. Tissue specimens were fixed in 4% paraformaldehyde solution and embedded in paraffin. The negative control sections were incubated with normal horse serum instead of the primary antibody and were processed together with the evaluated sections. The ovaries of multiparous pigs were used as a positive tissue control for the applied ERα or ERβ antibodies. The analytical procedure has been described previously (28).

#### Optical Density (Scanning) of Stained Slides

**Figure 1:**
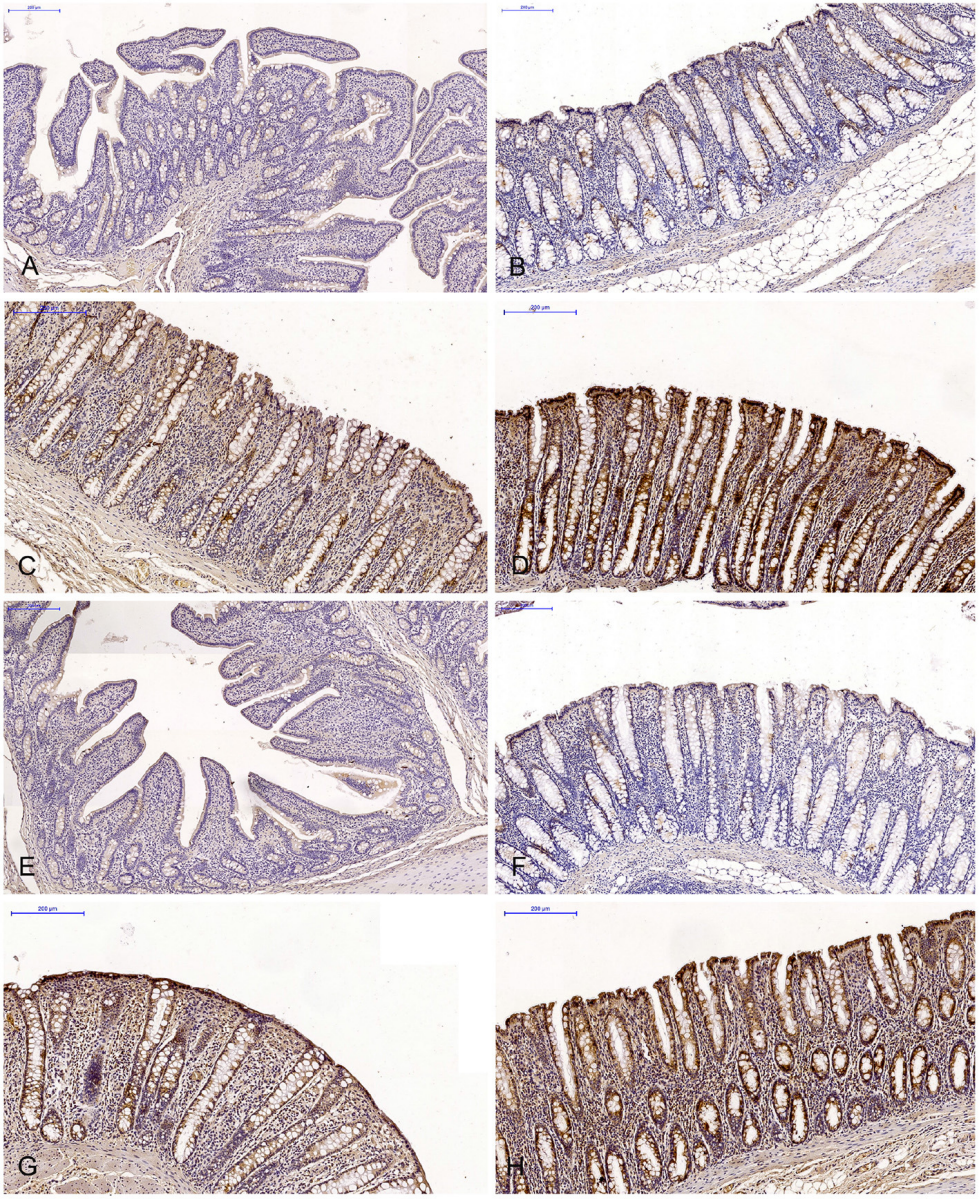
Scanned slides of immunohistochemical expression of ERα receptors in the descending colon in group C [**(A)** 0; **(B)** +; **(C)** ++; **(D)** +++] and group E [**(E)** 0; **(F)** +; **(G)** ++; **(H)** +++]. HE.

**Figure 2:**
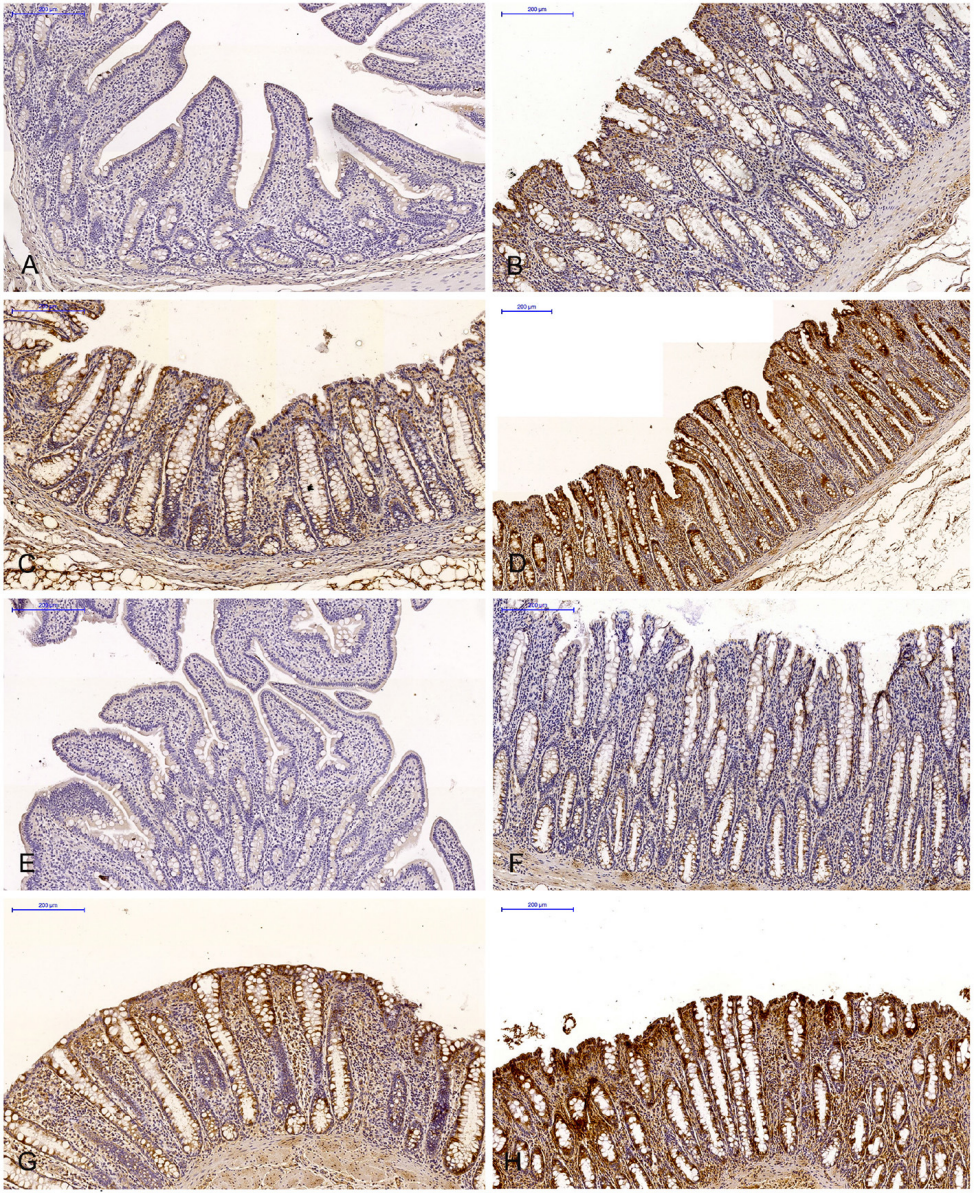
Scanned slides of immunohistochemical expression of ERβ receptors in the descending colon in group C [**(A)** 0; **(B)** +; **(C)** ++; **(D)** +++] and group E [**(E)** 0; **(F)** +; **(G)** ++; **(H)** +++]. HE.

The expression of ERα and ERβ in the intestinal samples collected from gilts in both groups was analyzed in scanned slides (Pannoramic MIDI Scanner, 3DHISTECH, Budapest, Hungary) with the use of NuclearQuant software (3DHISTECH, Hungary) (see Figures 1, 2). The slides were converted into digital images, and nuclear immunoreactivity was evaluated. The nucleus detection profile was as follows: radius – 1.50-2.10 μm, minimum nuclear area – 0.9 μm, minimum circularity – 3, smoothness – 1. ERα and ERβ expression was evaluated on a 4-point scale: negative = 0 (0 points), weak and homogeneous = + (1 point), mild or moderate and homogeneous = ++ (2 points), intense or strong and homogeneous = +++ (3 points). Staining intensity was evaluated on the following scale: from 0 – none of the below, + average intensity <190 (CD BrownInt), ++ average intensity <170 (CD BrownInt), +++ average intensity <100 (CD BrownInt) (29). The results were presented in terms of the average percentage of cells expressing ERα and ERβ. The presence of ER proteins was determined based on the value of P-ER which represents the average number of enterocytes showing nuclear expression of ER proteins in 10 fields of view for at least 1,000 cells. The analysis of optical density and the nuclear detection profile used was performed by the authors, and the results had been previously published in several papers (9, 28).

The results of immunohistochemical examinations of the expression of ERα and ERβ in the gastrointestinal tract of pre-pubertal gilts from both groups were analyzed statistically in two analytical approaches. In the first approach, the expression of both ER was analyzed statistically and compared between groups. In the second approach, the influence of sampling date (duration of exposure) on ER expression in each group and in a given intestinal segment was statistically analyzed. In both analytical approaches and in both groups, the effects of the 6-week exposure to DON on the expression of selected ER was evaluated in selected segments of the digestive tract on a 4-point scale (negative = 0 points; weak and homogeneous = 1 point; mild or moderate and homogeneous = 2 points; intense or strong and homogeneous = 3 points).

### Expression of *CYP1A1* and *GSTP1* Genes

#### Collection and Storage of Samples for RNA Extraction

Segments of the ascending colon and descending colon were collected immediately after cardiac arrest. The specimens were stored in RNA*later* solution (Sigma-Aldrich; Germany), in accordance with the manufacturer's instructions. The samples were collected on the same days of slaughter.

#### Total RNA Extraction and cDNA Synthesis

The total RNA was extracted (~20 mg per sample tissue; *n* = 3 in each group), and purified using a Total RNA Mini Isolation Kit (A&A Biotechnology; Poland). The final RNA concentration and purity was determined using a UV-Vis spectrophotometer (BioPhotometer, Eppendorf; Germany), obtaining A260/280 ratios between 1.70 and 1.86 in all the samples. Next, the samples were incubated with DNase I (Roche Diagnostics; Germany), and then used to synthesize cDNA using a RevertAid™ First Strand cDNA synthesis kit (Fermentas; Lithuania). Briefly, the reaction contained 1 μg of the DNAse-treated total RNA and 5 μM of oligo(dT)_18_ primer. After incubation at 65°C for 5 min, the samples were chilled on ice, and the following reagents were added in: 4 μL of 5× Reaction Buffer, 20 U of RiboLock™ RNAse Inhibitor, 1 mM of dNTP mix, and 200 U of RevertAid M-MuLV Reverse Transcriptase. The reaction was carried out at 42°C for 60 min, followed by heat-inactivation at 70°C for 5 min. Synthesized cDNA samples were stored at −80°C until used.

#### qPCR

Real-Time PCR primers for target mRNAs were designed using the Primer-BLAST tool (30) based on the reference species (Table 1). The real-time PCR assay was carried out in the ABI 7500 Real-Time PCR system thermocycler (Applied Biosystems; USA) in singleplex mode. Each PCR reaction tube contained 10 μL of the FastStart SYBR Green Master ROX mix (Roche Diagnostics), 0.25 (*CYP1A1*) or 0.5 (*GSTP1*) μM of each primer (forward and reverse; Table 1), 1 μL of previously synthesized cDNA as a template supplemented with PCR-grade H_2_O to a final volume of 20 μL. Further details of the analytical procedure have been described elsewhere (31).

**Table 1:** Real-time PCR primers used in this study.

**Primer**	**Sequence (5′ → 3′)**		**Final concentration (μM)**	**Amplicon length (bp)**	**Reference**
*CYP1A1*	Forward	cagagccgcagcagccaccttg	0.25	226	NM_214412.1
	Reverse	ggctcttgcccaaggtcagcac	0.25		
*GSTP1*	Forward	acctgcttcggattcaccag	0.5	178	EW651453.2
	Reverse	ctccagccacaaagccctta	0.5		

### Statistical Analysis

The activity of ERα and ERβ in the digestive tract of gilts, and the expression of *CYP1A1* and *GSTP1* genes in the ascending colon and the descending colon were presented as geometric mean values (±) SD for each group, relative to control group from the beginning of the experiment (control 0d). The results were analyzed using Statistica software (StatSoft Inc., USA). Mean values in the control and exposed groups were compared by repeated measures one-way ANOVA based on the DON dose administered to pre-pubertal gilts. If differences were noted between groups, Tukey's *post-hoc* test was used to determine which pairs of group means were significantly different. In ANOVA, group samples are drawn from normally distributed populations characterized by the same variance. Since the above assumptions were not met in all cases, the equality of group means was tested using the Kruskal-Wallis test of ranks and the multiple comparisons test in ANOVA. Non-parametric tests were used to test for differences in samples.

## Results

The experimental diets did not contain mycotoxins or their mycotoxin concentrations were below the sensitivity of the method (VBS, values below the sensitivity of the method). Clinical signs of DON mycotoxicosis were not observed during the experiment. The levels of modified or masked mycotoxins were not determined in the study. All laboratory and statistical analyses were carried out in 2013.

**Figure 3:**
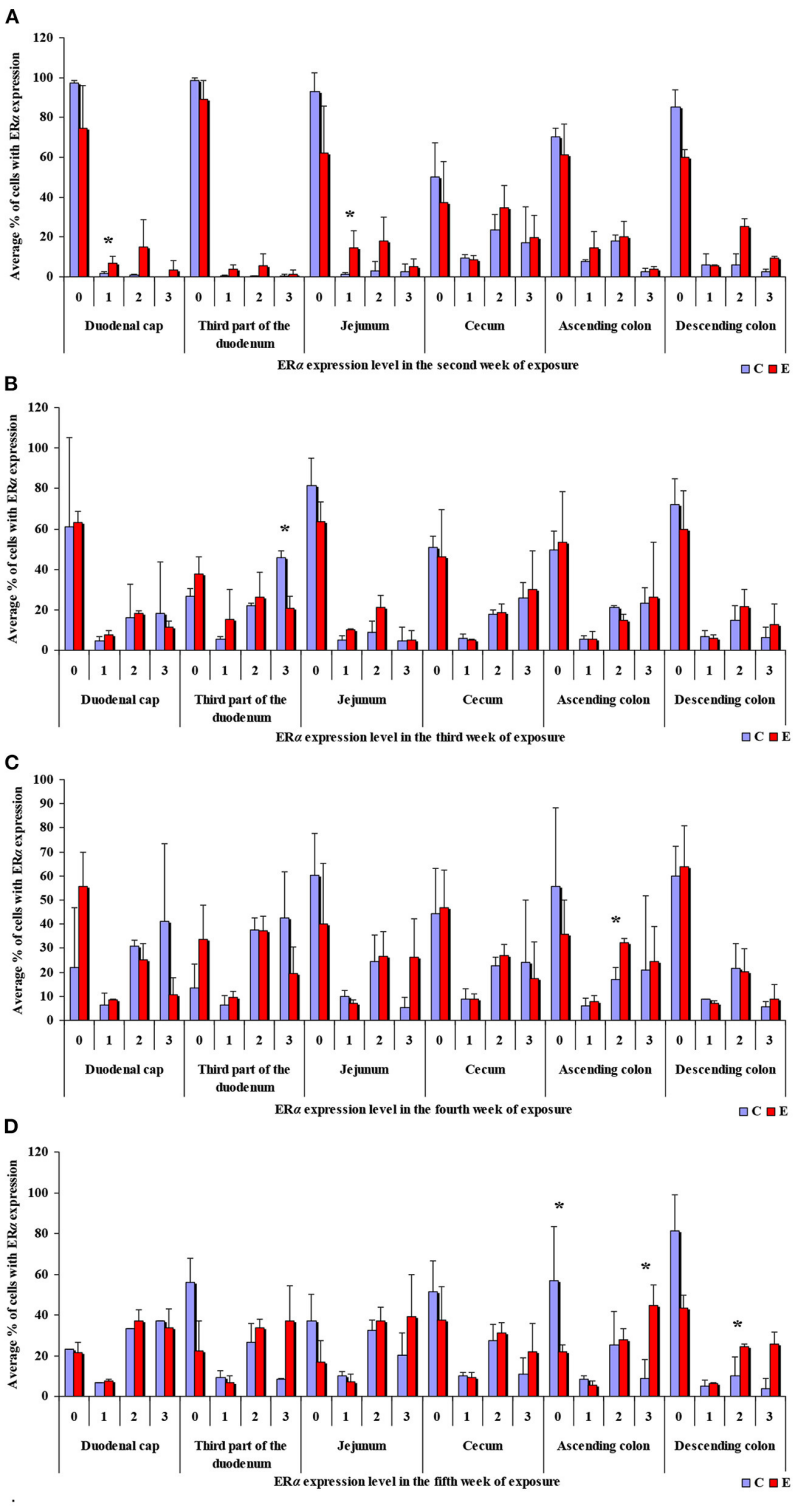
Immunohistochemical expression of ERα (graded on a 4-point scale: negative = 0 points; weak and homogeneous = 1 point; mild or moderate and homogeneous = 2 points; intense or strong and homogeneous = 3 points) in different intestinal segments in selected weeks of exposure: **(A)** in week II; **(B)** in week III; **(C)** in week IV; **(D)** in week V. ERα expression was presented as mean values (±) and standard deviation (SD) for each sample. *P ≤ 0.05 were compared with the control group.

**Figure 4:**
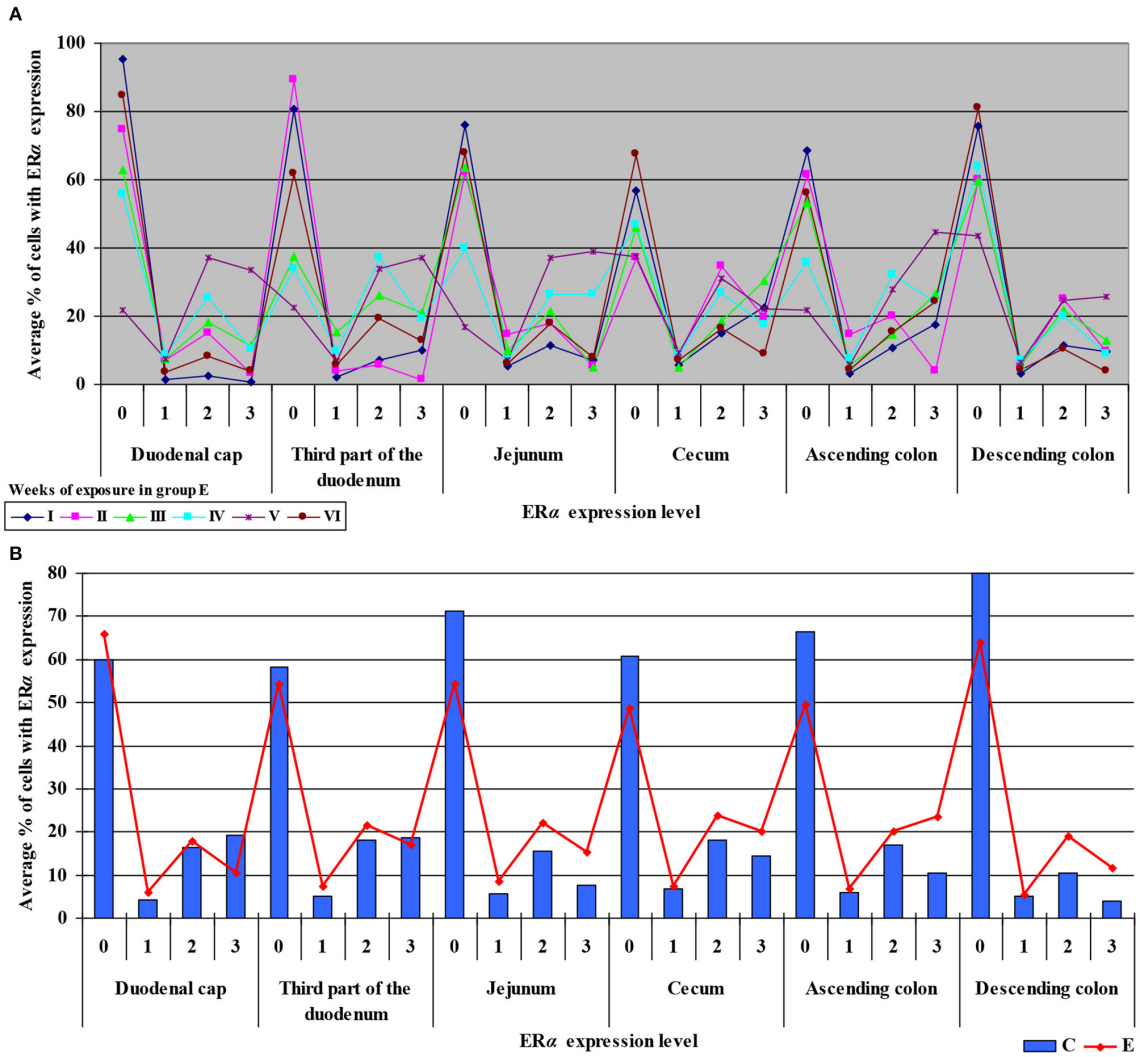
Immunohistochemical expression of ERα in the control and exposed groups (graded on a 4-point scale: negative = 0 points; weak and homogeneous = 1 point; mild or moderate and homogeneous = 2 points; intense or strong and homogeneous = 3 points) in different intestinal segments and in selected weeks of exposure: **(A)** in group E; **(B)** average expression values from all weeks of exposure in both groups.

**Figure 5:**
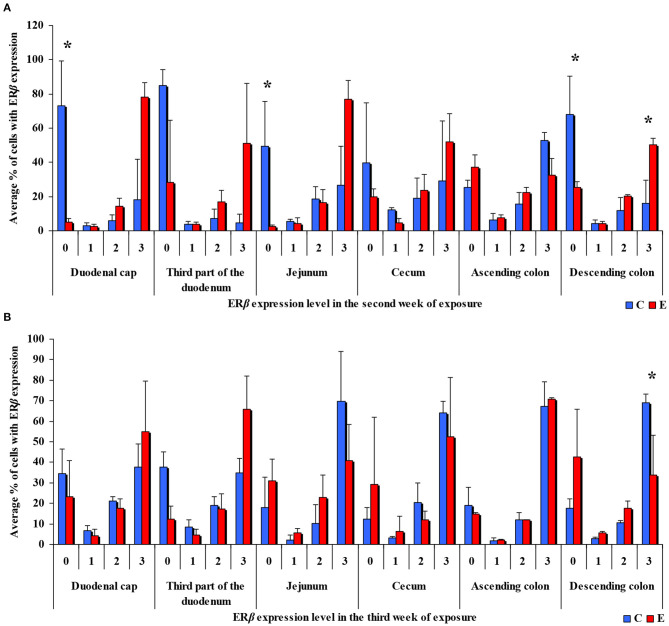
Immunohistochemical expression of ERβ (graded on a 4-point scale: negative = 0 points; weak and homogeneous = 1 point; mild or moderate and homogeneous = 2 points; intense or strong and homogeneous = 3 points) in different intestinal segments in selected weeks of exposure: **(A)** in week II; **(B)** in week III. ERβ expression was presented as mean values (±) and standard deviation (SD) for each sample. *P ≤ 0.05 were compared with the control group.

**Figure 6:**
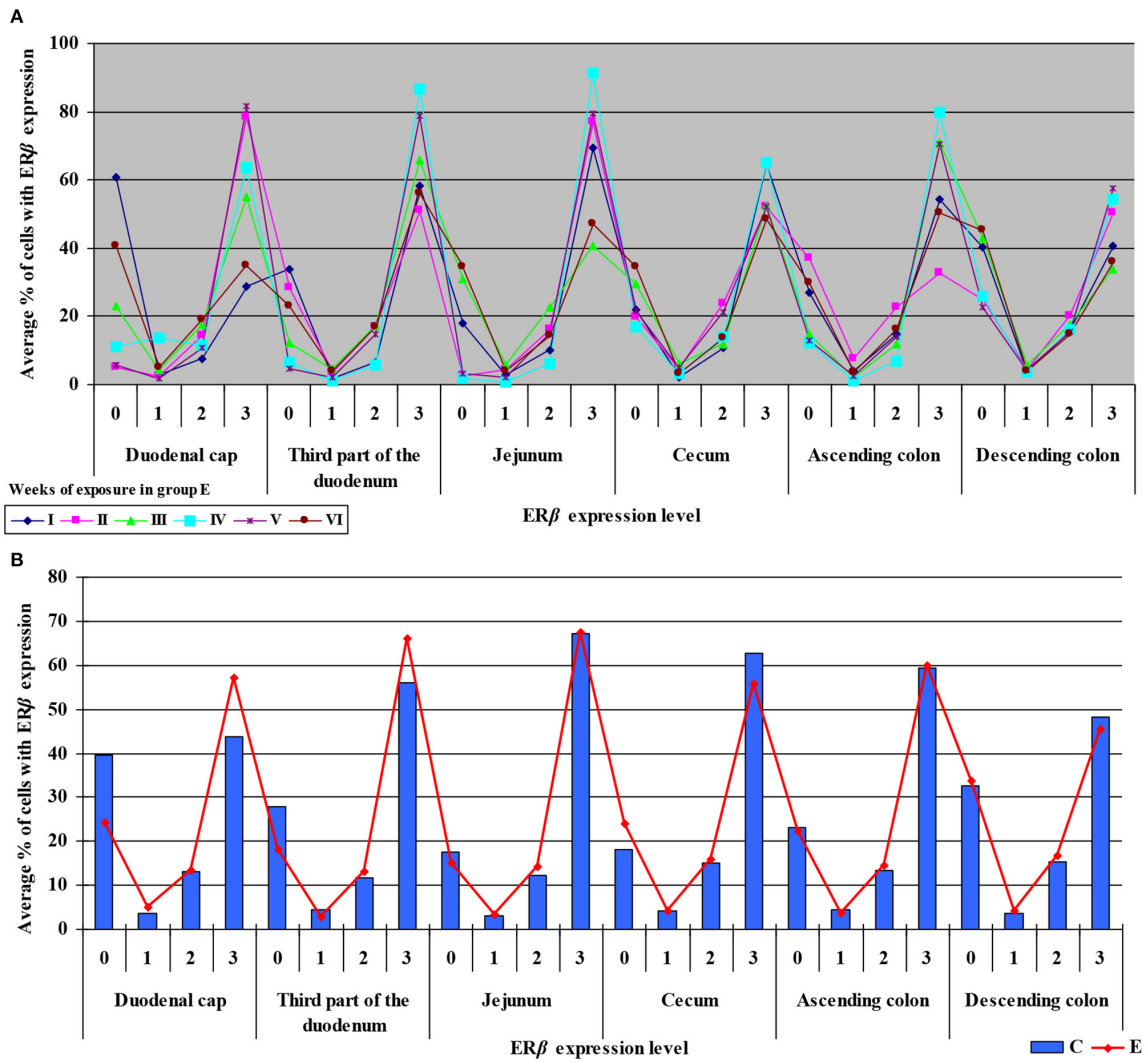
Immunohistochemical expression of ERβ in the control and exposed groups (graded on a 4-point scale: negative = 0 points; weak and homogeneous = 1 point; mild or moderate and homogeneous = 2 points; intense or strong and homogeneous = 3 points) in different intestinal segments and in selected weeks of exposure: **(A)** in group E; **(B)** average expression values from all weeks of exposure in both groups.

In the first analytical approach, the immunohistochemical expression of ERα and ERβ in each segment of the porcine gastrointestinal tract (where the samples were collected) was compared in all (six) weeks of exposure, between the control group (C) and the experimental group (E). In this analytical approach, ER expression was presented graphically (refer to Figures 3–**6**). The results were not presented graphically for the segments of the intestinal tract where statistical differences were not found (refer to Supplementary Figures 1–4).

In this analytical approach, no statistical differences in the immunohistochemical expression of ERα not shown were observed in weeks I and VI (see Supplementary Figures 1A,B). In week II (see Figure 3A), statistical differences in the immunohistochemical expression of ERα were noted in the duodenal cap and the jejunum, in week III (see Figure 3B), differences were found in the third part of duodenum, in week IV (see Figure 3C), differences were noted in the ascending colon, and descending colon in week V (see Figure 3D), differences were observed in the ascending colon.

The levels of ERα expression in different tissues and in different weeks of exposure were also presented graphically (see Figure 4 and Supplementary Figure 2).

The average percentage of cells expressing ERα was similar in both groups. The highest average percentage of cells expressing ERα was noted in unstained cells (0 points), in particular in group C, whereas the lowest average percentage of cells expressing ERα was observed in the strongly stained cells (3 points) in group C. In group E, the average percentage of unstained cells (0 points) was lower, whereas the average percentage of stained cells (1–3 points) was generally higher. These results indicate that the average percentage of strongly stained cells (3 points) expressing ERα increased steadily during exposure to DON.

In the same analytical approach (see Figure 5), differences in the immunohistochemical expression of ERβ were noted (at *P ≤ 0.05) in week II (see Figure 5A) in the duodenal cap, jejunum and the descending colon, and in the descending colon, and in week III (see Figure 5B), differences were found only in the descending colon. No significant differences in the immunohistochemical expression of ERβ were noted in weeks I, IV, V, or VI (see Supplementary Figures 3A–D).

Similarly, the levels of ERβ expression were presented in Figure 6 and Supplementary Figure 4. In general, no statistical differences in ERβ expression were found between the groups.

The average percentage of cells expressing ERβ was similar in both groups. ERβ expression continued to increase in intensely stained cells (3 points) on successive days of exposure to DON in both groups. In group E, a considerable increase in ERβ expression was observed in the duodenal cap and the third part of the duodenum (see Figure 6B).

**Figure 7:**
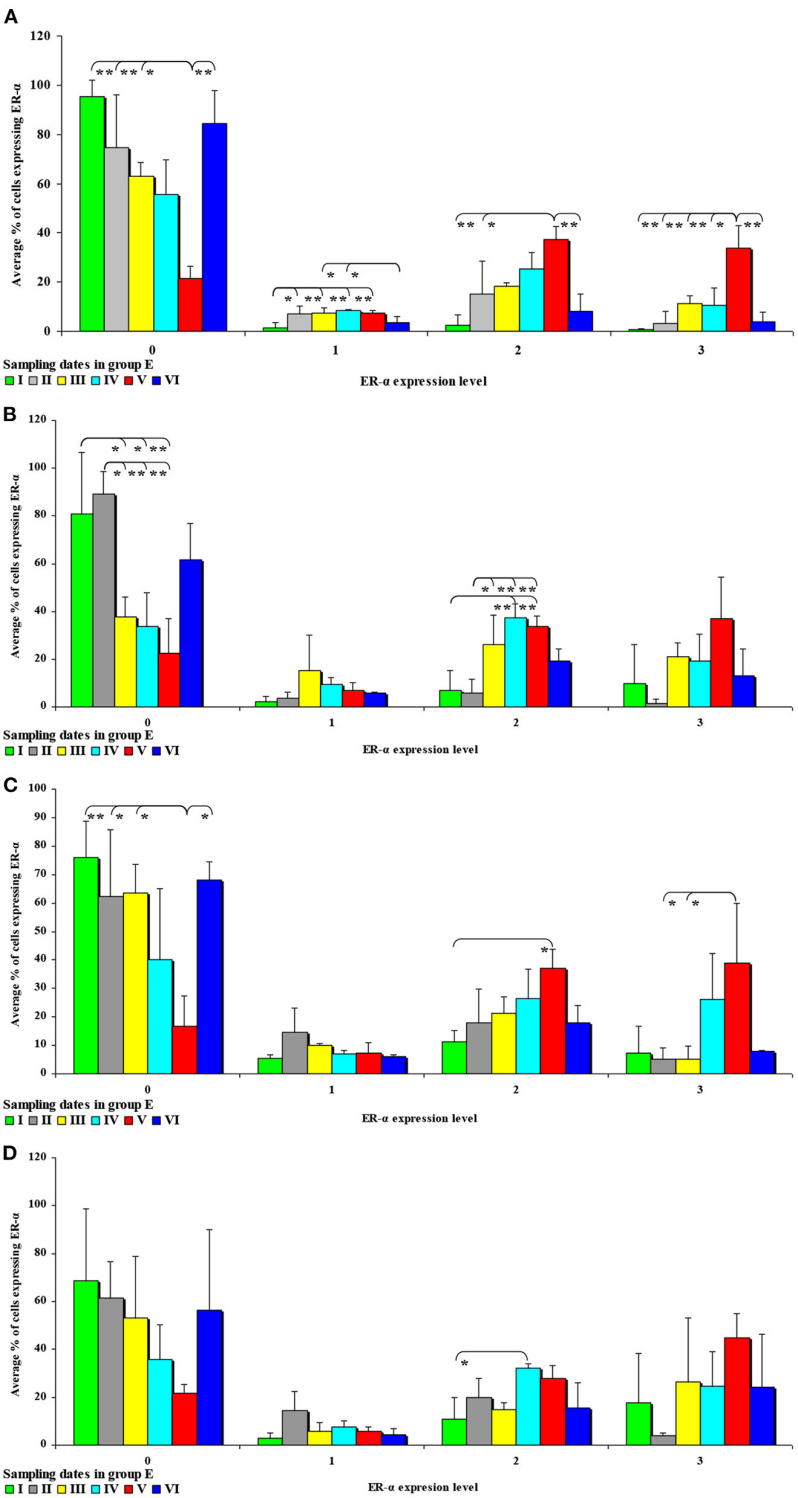
The effect of DON on the immunohistochemical expression of ERα (graded on a 4-point scale: negative = 0 points; weak and homogeneous = 1 point; mild or moderate and homogeneous = 2 points; intense or strong and homogeneous = 3 points) in the intestines of pre-pubertal gilts from **group E: (A)** in the duodenal cap in selected weeks of exposure; **(B)** in the third part of the duodenum in selected weeks of exposure; **(C)** in the jejunum in selected weeks of exposure; **(D)** in the ascending colon in selected weeks of exposure. ERα expression is presented as mean values (±) and standard deviation (SD) in selected samples. *P ≤ 0.05 and **P ≤ 0.01 compared with the remaining groups.

**Figure 8:**
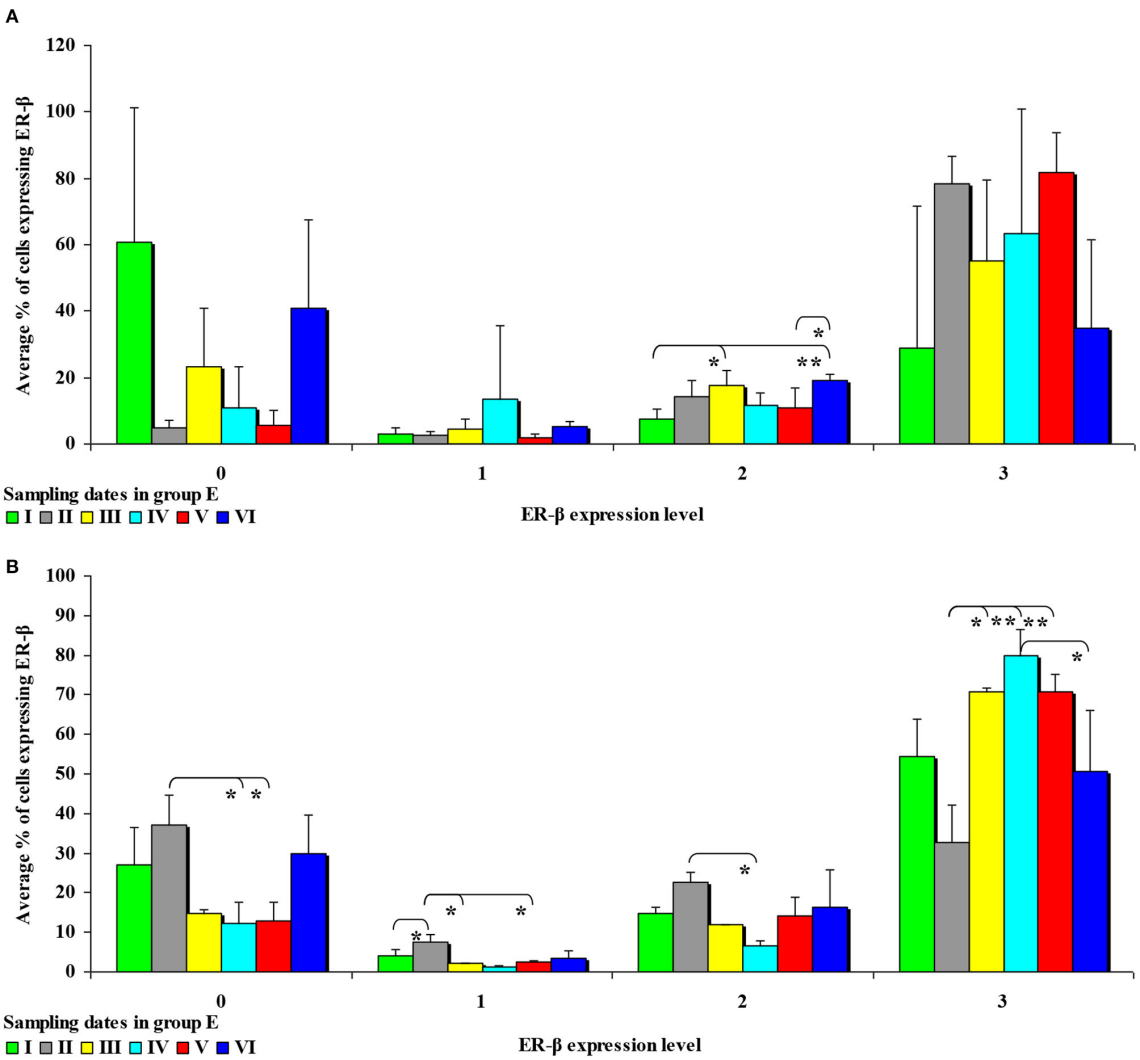
The effect of DON on the immunohistochemical expression of ERβ (graded on a 4-point scale: negative = 0 points; weak and homogeneous = 1 point; mild or moderate and homogeneous = 2 points; intense or strong and homogeneous = 3 points) in the intestines of pre-pubertal gilts from **group E: (A)** in the duodenal cap in selected weeks of exposure; **(B)** in the ascending colon in selected weeks of exposure. ERβ expression is presented as mean values (±) and standard deviation (SD) in selected samples. *P ≤ 0.05 and **P ≤ 0.01 compared with the remaining groups.

In the second analytical approach (evaluating the influence of exposure time on ER expression), ER expression was presented only graphically due to a large number of significant differences (refer to Figures 7, 8). The results were not presented graphically for the segments of the intestinal tract where significant differences were not found (refer to Supplementary Figures 5, 6).

In this analytical approach, no significant differences in the immunohistochemical expression of ERα were found in the descending colon in group C and in the cecum and the descending colon in group E.

An analysis of the results presented in Supplementary Figure 5 indicates that the immunohistochemical expression of ERα continued to decrease in unstained (negative and homogeneous, 0 points) cells and in weakly stained and homogeneous cells (1 point) on successive sampling dates and in successive intestinal segments. An increase in ERα expression in intestinal cells [moderately (2 points) and strongly (3 points) stained cells] appears to be a normal process in pre-pubertal gilts.

In group E (Figure 7), the percentage of unstained (negative and homogeneous, 0 points) cells also decreased, whereas the percentage of cells showing mild and homogeneous (2 points) and strong and homogeneous (3 points) expression of ERα continued to increase. These findings indicate that feed contamination with low doses of DON increases the percentage of intestinal cells expressing ERα (2 points and 3 points) in pre-pubertal gilts.

In this analytical approach, no significant differences in the immunohistochemical expression of ERβ were noted in the cecum and the ascending colon in group C, and in the duodenal cap, the third part of the duodenum, the cecum and the descending colon in group E.

It can be assumed that the observed ERβ expression levels in the intestinal cells of control group animals (Supplementary Figure 6) are normal (physiological) and typical of pre-pubertal gilts. In proximal intestinal segments, the percentage of unstained (negative and homogeneous, 0 points) cells expressing ERβ continued to decrease on successive days of exposure. In turn, the percentage of cells where ERβ expression was strong and homogeneous (3 points) increased during the experiment. These differences in ERβ expression levels disrupt the stability of endocrine processes.

Significant differences were noted only in the duodenum (in the proximal segment) and in the descending colon (Figure 8). The presence of DON in feed increased the percentage of intestinal cells strongly expressing ERβ (3 points) from 45% in the duodenum on sampling date I to 79% in the jejunum on sampling date IV on average. More stable ERβ expression may have positive implications for endocrine processes in pre-pubertal gilts.

The ER expression index should be calculated for each receptor type to obtain prognostic information. The results can be used to determine the degree/direction of expression of both types of analyzed ER. The ER expression index (P-ER) was calculated in 576 samples. The mean values of P-ER reached 66 ± 10 for ERα and 26 ± 11 for ERβ in group C, and 56 ± 9 for ERα and 23 ± 7 for ERβ in group E. P-ER values were not normally distributed (Table 2).

**Table 2:** ER expression at various absorption levels during the entire experiment in the analyzed segments of the gastrointestinal tract in pre-pubertal gilts.

**Group**	**Degree of ER expression**	**Duodenal cap**	**Third part of duodenum**	**Jejunum**	**Cecum**	**Ascending colon**	**Descending colon**
**ER*****α***							
Group C	0	A	A	C	A	C	D
	1	A	B	C	D	C	B
	2	C	D	B	D	C	A
	3	D	D	A	C	B	A
Group E	0	D	B	B	A	A	D
	1	A	C	D	C	B	A
	2	A	C	C	D	B	A
	3	A	C	B	D	D	A
**ER*****β***							
Group C	0	D	C	A	A	B	C
	1	B	D	A	D	D	B
	2	B	A	A	D	B	D
	3	A	C	D	D	C	A
Group E	0	C	A	A	C	B	D
	1	D	A	A	C	B	C
	2	A	A	B	D	B	D
	3	B	D	D	B	C	A

*The values of P-ERs were divided into four groups and presented for each group: very low (A), low (B), high (C) and very high (D)*.

In group C, the average value of P-ERα was determined at 66, reaching 62 and 73 in the lower and upper quartile, respectively. Based on the median, the upper and lower quartiles, the expression values were divided into four sub-groups: A – very low P-ERα (P-ERα <62), B–low P-ERα (62 < P-ERα <66), C–high P-ERα (66 < P-ERα <73), and D – very high P-ERα (P-ERα > 73) (Table 2). In group C, very low (A), low (B), high (C) and very high (D) values of P-ERα were noted in 7 (29%), 4 (17%), 7 (29%) and 6 (25%) cases, respectively. The statistical analysis, conducted for the mean, median, upper and lower quartile cut-off points, revealed no significant differences.

In group E, the average value of P-ERα was determined at 56, reaching 52 and 61 in the lower and upper quartile, respectively. Based on the median, the upper and lower quartiles, the expression values were divided into four sub-groups: A – very low P-ERα (P-ERα < 52), B–low P-ERα (52 ≤ P-ERα < 56), C–high P-ERα (56 < P-ERα < 61), and D – very high P-ERα (P-ERα > 61) (Table 2). In group E, very low (A), low (B), high (C) and very high (D) values of P-ERα were noted in 8 (33%), 5 (21%), 5 (21%), and 6 (25%) cases, respectively. The statistical analysis, conducted for the mean, median, upper and lower quartile cut-off points, revealed no significant differences.

In group C, the average value of P-ERβ was determined at 26, reaching 22 and 33 in the lower and upper quartile, respectively. Based on the median, the upper and lower quartiles, the expression values were divided into four sub-groups: A – very low P-ERβ (P-ERβ < 22), B–low P-ERβ (22 < P-ERβ < 26), C–high P-ERβ (26 < P-ERβ < 33), and D–very high P-ERβ (P-ERβ > 33) (Table 2). In group C, very low (A), low (B), high (C) and very high (D) values of P-ERβ were noted in 7 (29%), 5 (21%), 4 (17%) and 8 (33%) cases, respectively. The statistical analysis, conducted for the mean, median, upper and lower quartile cut-off points, revealed no significant differences.

In group E, the average value of P-ERβ was determined at 23, reaching 19 and 28 in the lower and upper quartile, respectively. Based on the median, the upper and lower quartiles, the expression values were divided into four sub-groups: A – very low P-ERβ (P-ERβ < 19), B–low P-ERβ (19 < P-ERβ < 23), C–high P-ERβ (23 < P-ERβ < 28), and D – very high P-ERβ (P-ERβ > 28) (Table 2). In group E, very low (A), low (B), high (C) and very high (D) values of P- P-ERβ were noted in 7 (29%), 6 (25%), 5 (21%) and 6 (25%) cases, respectively. The statistical analysis, conducted for the mean, median, upper and lower quartile cut-off points, revealed no significant differences.

**Figure 9:**
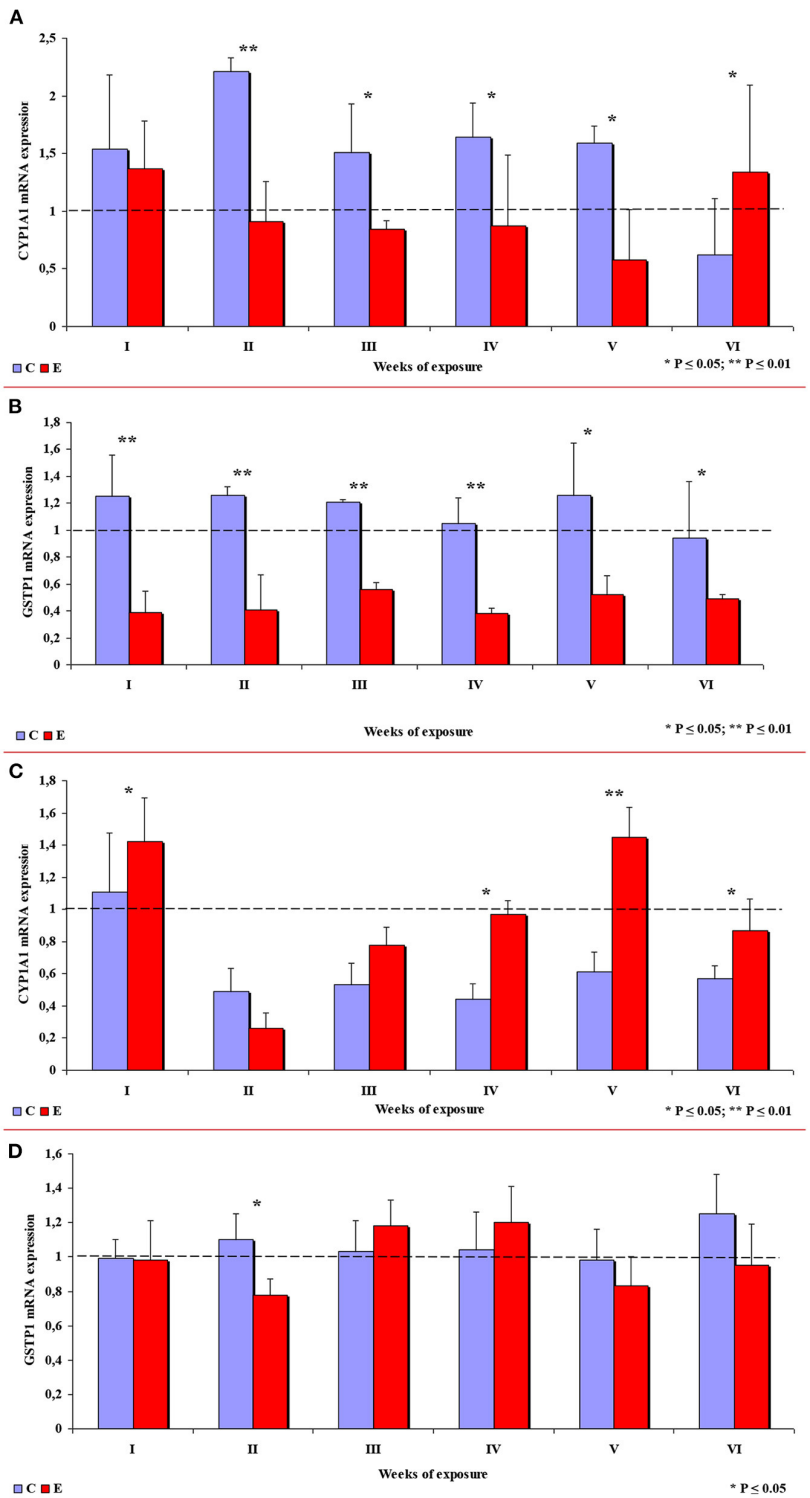
The expression of selected enzymes in the colon in different weeks of exposure: **(A)** *CYP1A1 m*RNA expression in the ascending colon; **(B)** *GSTP1 m*RNA expression in the ascending colon; **(C)** *CYP1A1 m*RNA expression in the descending colon; **(D)** *GSTP1 m*RNA expression in the descending colon. The bars represent mean values (*n* = 3) of enzyme gene expression ratios (ER ± SE) relative to a control sample at the beginning of the experiment (ER = 1.00; dashed line). Symbols (*) indicate experimental (E) groups that showed significantly different *m*RNA levels compared to a respective control (C) group (*P ≤ 0.05, **P ≤ 0.01).

In group E, the expression of the *CYP1A1* gene in the ascending colon decreased in comparison with group C, which is related to inflammation (32), except in week VI when *CYP1A1* expression was significantly higher in group E. In week II, the observed difference was highly significant, and significant differences were found in weeks III, IV and V (see Figure 9A). In group E, a highly significant decrease in the expression of the *GSTP1* gene was observed in weeks I to IV, and a significant decrease was noted in weeks V and VI (see Figure 9B).

In the descending colon, the expression of the *CYP1A1* gene was significantly higher in group E in weeks I, IV and VI, and highly significantly higher in week V (see Figure 9C). Significant differences in the expression of the *GSTP1* gene (higher values in group C) were observed only in week II. In the remaining weeks of the study, the mean values of this parameter fluctuated in both groups, but no significant differences were found (see Figure 9D).

## Discussion

### Estrogen Receptors

There is increasing evidence to suggest that mycotoxins and their metabolites [not only mycoestrogens–(33)] are directly involved in interactions that disrupt hormonal homeostasis [including steroid hormones, as demonstrated *in vitro* by–(1)]. These interactions are also mediated by ER. One of the important questions that needs to be answered is whether DON, an undesirable substance of natural origin, poses potential health risks in humans (2, 24, 34) and animals (22).

#### Optical Density

##### First Analytical Approach

No differences in the expression of both ER were found in the first and last week of the experiment, which could have several explanations.

The results of this study (see Figures 3–6) can be divided into two parts, corresponding to the analyzed intestinal segments. Firstly, DON undergoes relatively rapid biotransformation (16, 35, 36) in the proximal intestinal segments (both segments of the duodenum and jejunum), regardless of the week of exposure, or a counter-response (no response) of the macroorganism to the presence of a small dose of an undesirable substance is observed (33). The absence of significant differences on the first sampling date could be attributed to a very low mycotoxin dose that is “ignored” by the body. This assumption is partially confirmed by other studies (37), and it is consistent with the Tregs theory (38) which postulates that the expression of Tregs cells is not induced when the number of infectious factors is very low. It is also possible that DON comes into short-lived contact with ER in enterocytes, or does not come into such contact at all, because the mycotoxin is too rapidly absorbed by the macroorganism. Similar conclusions were formulated in another study that investigated a different mycotoxin (23). The above could also be due to the fact that DON concentrations are initially low, and the process of tissue saturation with the mycotoxin begins only in the second week of exposure (39, 40).

Secondly, ERα expression in the descending colon in group C reached nearly 100% in week I, and it was lowest (60%) in week IV when a negative value was noted (see Supplementary Figure 2). In group E, ERα expression was mostly intense or strong and homogeneous (3 points), ranging from 80% in weeks I and VI, through 60% in week IV to 42% in week V (see Figure 4A). The above could result from a compensatory mechanism (41), where the activity of low mycotoxin doses is suppressed and initial functions are restored despite ongoing exposure. According to an *in vitro* study conducted by Woelflingseder et al. (42), the compensatory mechanism may interfere with the glutathione-depleting effect under prolonged exposure to DON, which can potentially reduce the cytotoxic effect. On the other hand, in the present study, *GSTP1 m*RNA expression in both segments of the colon (see Figure 9), in particular the ascending colon (see Figure 9B), decreased in group E. The above could decrease the proliferation of epithelial cells (16), which promotes apoptosis, leading to controlled proliferation (43).

Therefore, the observed changes could be attributed to compensation as well as chemical stress resulting from exposure to DON (20).

##### Second Analytical Approach

This analytical approach confirms the observations made by Demaegdt et al. (1) that DON is a partial agonist of ERα. In this study, the immunohistochemical expression of ERα was mild or moderate and homogeneous (2 points) and intense or strong and homogeneous (3 points). The differences in the percentage of ERα-expressing cells were considerable (10% to 15% on average), but not statistically significant, and they were noted mainly in weeks III, IV and V (see Figure 7) in the duodenal cap, the third part of the duodenum, the jejunum and the ascending colon. These observations could be attributed to prolonged exposure to DON which disrupts physiological processes (8) and cell signaling and, consequently, leads to the inhibition of protein and nucleic acid synthesis, and disruption of steroidogenesis caused by cytochrome P450 (2). Steroidogenesis processes occur in tissues where steroidogenic enzymes are expressed, including the gastrointestinal tract (44). The disrupting effects of DON are limited to local tissues (16), as confirmed by the decrease in *CYP1A1 m*RNA expression in the ascending colon (see Figure 9A), but not in the descending colon in group E.

In the second analytical approach, the immunohistochemical expression of ERβ in group C was mainly negative (0 points) and intense or strong and homogeneous (3 points) (see Supplementary Figure 6). Negative expression was noted mainly in weeks II and I. Intense or strong and homogeneous expression (3 points) was observed mainly in weeks IV, V and VI in the duodenal cap, the third part of the duodenum, the jejunum and the descending colon. Considerable changes were noted in group E (see Figure 8): significant differences were found only in the duodenal cap and the ascending colon where ERβ expression was evaluated as intense or strong and homogeneous (3 points); a general decrease in expression processes was also noted in this group. In the highest percentage of ERβ-expressing cells, ERβ expression was also intense or strong and homogeneous (3 points) (see Figure 8B). An analysis of the results noted on each sampling date revealed that DON was accumulated until week V, particularly in the ascending colon. This specific situation observed at the end of the experiment may be attributed to the transactivation of ERβ (45), which could be due to intensified apoptosis (46) and proliferation in intestinal tissues as a result of Ying/Yang interactions (47). These findings could also be explained by the absence of competition (absence of ERα) in selected tissues [such as the colonic mucosa–(48)] whose functions are not affected by ERα. The colon is one of the key organs targeted by DON, which is why the observations made in this region are most pronounced. These processes are accompanied by histological changes which have been described by Pinton and Oswald (49) and Przybylska-Gornowicz et al. (50), and which take place mainly in the mucosa. Non-genomic factors that modulate regulatory membrane proteins could also play a role (47, 51).

In the second analytical approach, enhanced expression of ERβ under exposure to a low DON dose was a biological effect that inhibited ERα-related functions (52). A comparison of both analytical approaches with previous findings (9) indicates that the immunohistochemical expression of ER proceeds differently, which could be attributed to the fact that the cited study examined the effects of mixed mycotoxicosis (DON/ZEN). The exposure to both DON and ZEN increased ERα expression in the jejunum and the ascending colon, but expression levels in these intestinal segments continued to decrease during exposure. The expression of ERß decreased steadily during exposure, in particular in the cecum and the descending colon. The expression of ERß continued to increase in the remaining intestinal segments. These observations suggest that during mixed (DON/ZEN) mycotoxicosis, ERß plays a dominant role in the intestinal mucosa of pre-pubertal gilts. A comparison of previous findings (9) with the results of the present study indicates that at low concentration, DON acts as a partial agonist that only partially activates the receptors.

#### ER Expression Index

The ER index was calculated by comparing P-ERα/P-ERβ because both ER occur in various proportions. An increase in the ER index points to enhanced proliferative processes resulting from higher expression of ERα. A decrease in the ER index suggests that steroidogenesis processes were inhibited and that ERβ expression was enhanced. The ER expression index (P-ERα/P-ERβ) was calculated to determine whether DON is a potential disruptor of hormonal homeostasis at the level of nuclear receptor signaling or, in other words, whether DON can act as an exogenous ligand for both ERα and ERβ. The values of the ER expression index were higher in group E between the cecum and the descending colon. However, the graphic presentation of ER expression (see Figure 4B) revealed that ERα expression decreased whereas ERβ expression remained at a similar level in both groups (see Figure 6B). The slight differences between groups indicate that DON is a relatively weak agonist *in vivo*, but it is not a weak antagonist of ER as suggested by Demaegdt et al. (1). This minor difference in activity could lead to hormonal activation resulting from the fact that plant materials generally contain much higher levels of DON than other *Fusarium* mycotoxins (6), which suggests that the bioavailability of DON increases during exposure (16).

The fact that DON is capable of inducing the expression of ERβ at the same level as in group C (see Figure 6B) which is more abundant in intestinal walls (47), indicates that this mycotoxin can participate in steroidogenesis (2) and enhances antioxidant activity. The latter observation is justified by the fact that both ER mediate estrogen activity. An important question that should be answered is whether DON can be regarded as an antioxidant solely because it induces the expression of ER. At the same time, DON inhibits antioxidant enzymes, thus increasing the production of ROS (45), and decreasing intracellular GSH levels (42). In the present study, *GSTP1 m*RNA expression decreased in the colon (see Supplementary Figures 6B,D) (excluding in the descending colon in weeks III and IV–see Supplementary Figure 6D), the immunohistochemical expression of ERα decreased and ERβ expression remained unchanged.

The maintenance of ERβ expression (see Figure 6B) and supression of ERα (see Figure 4B) may lead to the stimulation of cell proliferation and promotion of apoptosis (47). The above suggests that the presence of DON in feed can offer protection against selected hormone-sensitive tumors and neoplastic changes in the large intestine in mammals.

### *m*RNA Expression of Genes Encoding Selected Enzymes in the Colon

Unlike some other mycotoxins, DON is not biologically transformed into more toxic compounds and is not oxidized to less toxic compounds during phase I biotransformation (16). Various DON inactivation mechanisms have been identified in phase II biotransformation. One of these mechanisms involves the induction of GSTπ subfamilies, which suggests that DON can act as a substrate for GST during phase II biotransformation (53). A different hypothesis postulates that GST activity increases due to the oxidative stress induced by DON (53).

The absence of definitive answers to the above questions was the key motivation for investigating the effect of DON on the activity of two enzymes, CYP1A1 and GSTP1, which play important roles in the biotransformation of *Fusarium* mycotoxins.

#### CYP1A1 Metabolic Enzyme

A review of the literature indicates that DON disrupts cell signaling in mammals (34) and exerts multidirectional effects by distorting gastrointestinal homeostasis and steroidogenesis with the involvement of cytochrome P450 (CYP) (2, 44). Some of CYP enzymes, e.g., CYP1A1, are involved in the conversion of procarcinogens to carcinogens (16, 17). Transcriptional activation of the *CYP1A1* gene can take place under exposure to undesirable substances such as DON (16, 34, 44) or phytoestrogens (18). Detoxifying effects can be achieved by suppressing the activity of the CYP1A1 enzyme (22). This type of activity was detected in the ascending colon on nearly all sampling dates (see Figure 9A), excluding week VI. The results noted on the last sampling date are difficult to interpret because expression levels were higher in group E. It has been hypothesized that CYP1A1 alleles may increase the risk of cancer because they encode enzymes with higher metabolic activity (54). However, it should be noted that the accumulation of DON in tissues (22) increased in successive weeks of exposure. The above suggests that biotransformation processes do not evolve continuously during exposure, and the carryover factor increased from 0.012–0.022 in weeks I to IV to 0.032–0.049 in the last week of the experiment. The above interpretation does not apply to the descending colon (see Figure 9C), where the carry-over of DON was detected only in week IV. These results can be extrapolated to suggest that the “transient dysbiosis” of the gut microbiota plays a key role in the biotransformation of DON, in particular in the large intestine of pre-pubertal gilts (40–56). However, further research is needed to validate this assumption in all mammals.

Considerable differences in *CYP1A1 m*RNA expression were found between the ascending colon and the descending colon, which complicates the interpretation of the results. The presence of DON in ingested feed inhibited gene expression in the ascending colon, which suggests that this mycotoxin can contribute to preventing steroid-dependent carcinogenesis. In the descending colon, DON increased gene expression, which, together with the presence of endogenous factors in the intestinal lumen, points to an increase in the enzyme's detoxifying activity.

#### GSTP1 Gene Encoding Phase II Metabolic Enzymes

In the current study, an attempt was also made to determine whether prolonged exposure to a low dose of DON modifies *GSTP1 m*RNA expression and the associated processes in pre-pubertal gilts. According to Woelflingseder et al. (42), the *in vitro* modulatory effects of GSH on DON toxicity led to only a minor suppression of proliferative processes and cytotoxic effects. In the present study, *GSTP1 m*RNA expression decreased steadily in the ascending colon during the experiment (see Figure 9B). However, in the descending colon, *GSTP1 m*RNA expression was higher in group E in weeks III and IV (see Figure 9D). These observations could be attributed to: (i) balanced demand and supply (homeostasis) of *GSTP1 m*RNA which is required for the maintenance of cytoprotective and detoxifying functions during phase II biotransformation (20); (ii) excessive saturation with intracellular GSH in the ascending colon and the descending colon in response to the overexpression of *GSTP1 m*RNA (19); (iii) intensified transactivation of ERβ (45); or (iv) intensified apoptosis (34, 46) and/or controlled proliferation in intestinal tissues (34, 47). These observations are consistent with the results reported *in vitro* (42).

The exposure to a low mycotoxin dose led to a controlled decrease in *GSTP1 m*RNA expression, and a consensus was maintained between intestinal cells, the degree of exposure to an undesirable substance and the detoxifying effect.

### Summary

The aim of this study was to determine *in vivo* the effect of DON, administered *per os* at 12 μg/kg BW to pre-pubertal gilts for 42 days, on the immunohistochemical expression of intestinal ERs and the expression of genes encoding *CYP1A1 m*RNA and *GSTP1 m*RNA in the colon on different sampling dates. The research findings indicate that exposure to the analyzed DON dose led to (i) a decrease in ERα expression by ~15% between the jejunum and the descending colon; (ii) an increase in ERβ expression in the duodenum, and the maintenance of its expression at the same level as in group C in the remaining intestinal segments; (iii) a significant decrease in *CYP1A1 m*RNA expression in the ascending colon, and a significant increase in its expression in the descending colon; and (iv) a decrease in *GSTP1 m*RNA expression in the colon on most sampling dates.

To the best of the authors' knowledge, the is the first study to indirectly demonstrate that the presence of the analyzed DON dose in feed (12 μg DON/kg BW *per os* for a period 42 days) probably offers protection against uncontrolled proliferation in the large intestine of pre-pubertal gilts, and contributes to maintaining a stable consensus between the degree of exposure to DON and the detoxifying effect in mammals.

## Data Availability Statement

The original contributions presented in the study are included in the article/Supplementary Material, further inquiries can be directed to the corresponding author/s.

## Ethics Statement

All procedures in this study were carried out in compliance with Polish legal regulations for the determination of the terms and methods for performing experiments on animals and with the European Community Directive for the ethical use of experimental animals. The protocol was approved by the Local Ethical Council in Olsztyn (opinion No. 88/N of 16 December, 2009).

## Author Contributions

MG, ŁZ, and MTG designed the experiments. The data were analyzed and interpreted by MG, IO-D, PB, and SL-Ż. The manuscript was drafted and critically read by MG, ŁZ, and MTG. The manuscript was revised, read, and approved by all authors.

## Conflict of Interest

The authors declare that the research was conducted in the absence of any commercial or financial relationships that could be construed as a potential conflict of interest.

## Abbreviations

BW: body weight
C: control group
Cq: quantitative cycle
*CYP1A1*: cytochrome P450 1A1 gene
E: exposed group
EDs: endocrine disruptors
ERs: estrogen receptors
ERα: estrogen receptor alpha
ERβ: estrogen receptor beta
GSH: glutathione
*GSTP1*: glutathione S-transferase π gene
NOAEL: no-observed-adverse-effect level
P-ER: ER expression index
Real-Time PCR: real-time polymerase chain reaction
SD: standard deviation.
