# Supplementary material for: The Effect of 42-Day Exposure to a Low Deoxynivalenol Dose on the Immunohistochemical Expression of Intestinal ERs and the Activation of CYP1A1 and GSTP1 Genes in the Large Intestine of Pre-pubertal Gilts
Source: Front Vet Sci. 2021 Jul 19;8:644549. doi: 10.3389/fvets.2021.644549 (PMC8326516; doi:10.3389/fvets.2021.644549)
Supplement: Supplementary file 1 [file Data_Sheet_1.pdf]

Supplementary materials

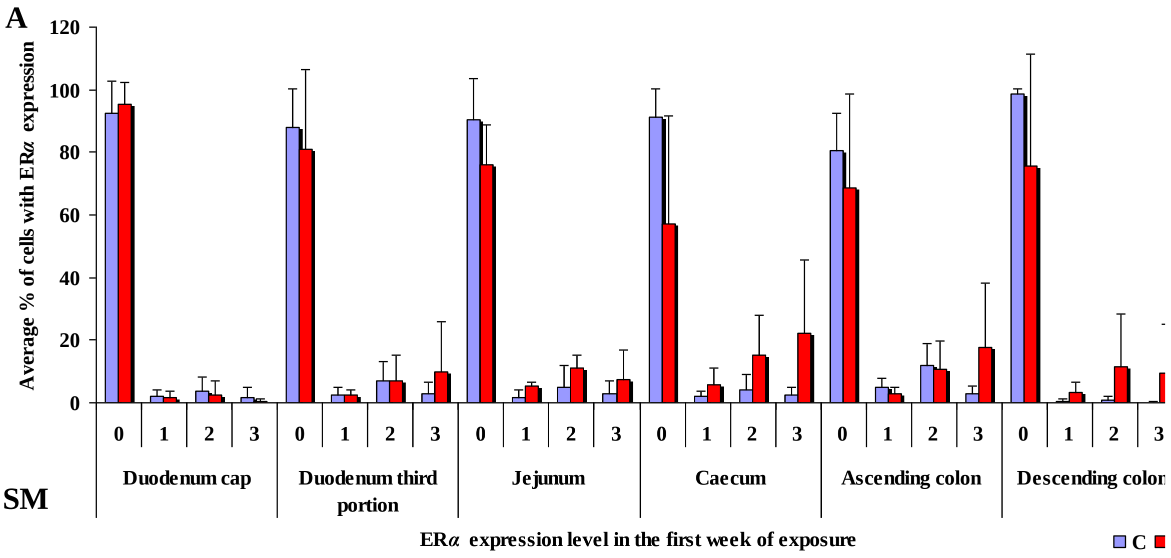

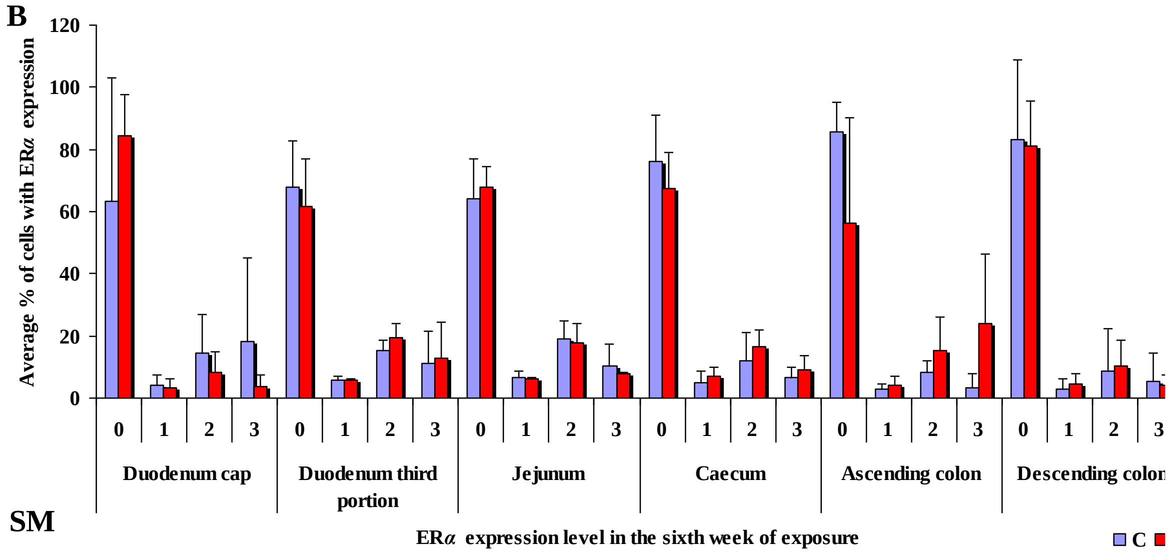

FIGURE SM 1│Immunohistochemical expression of ERα (graded on a 4-point scale: negative = 0 points; weak and homogeneous = 1 point; mild or moderate and homogeneous = 2 points; intense or strong and homogeneous = 3 points) in different intestinal segments in selected weeks of exposure: A – in week I; B – in week VI. ERα expression was presented as mean values (±) and standard deviation (SD) for each sample. *P ≤ 0.05 were compared with the control group.

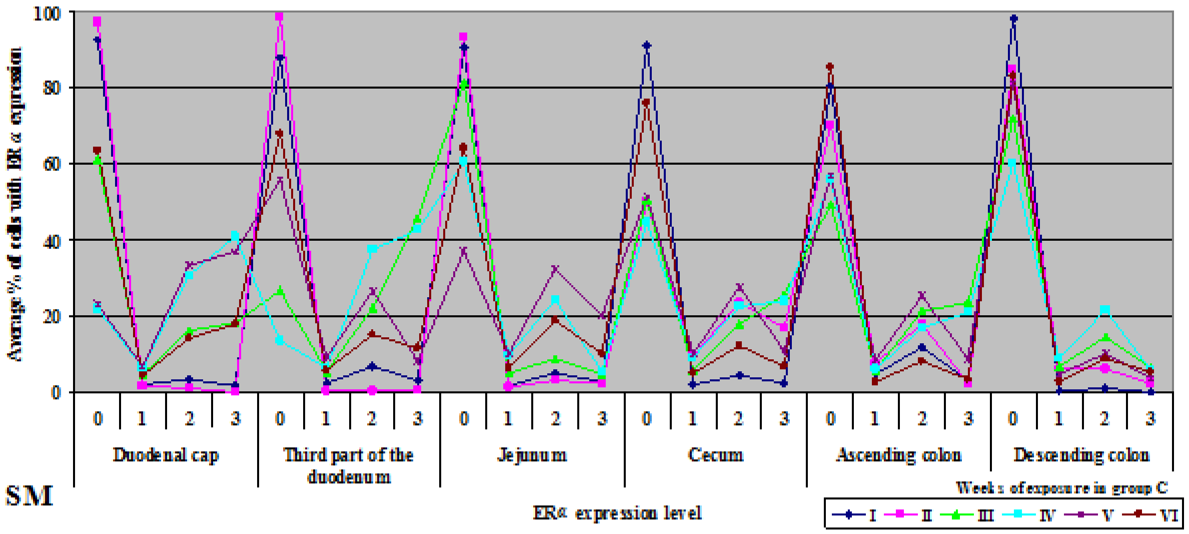

FIGURE SM 2 | Immunohistochemical expression of ERα in the control group (graded on a 4-point scale: negative = 0 points; weak and homogeneous = 1 point; mild or moderate and homogeneous = 2 points; intense or strong and homogeneous = 3 points) in different intestinal segments and in selected weeks of exposure - average expression values from all weeks of exposure.

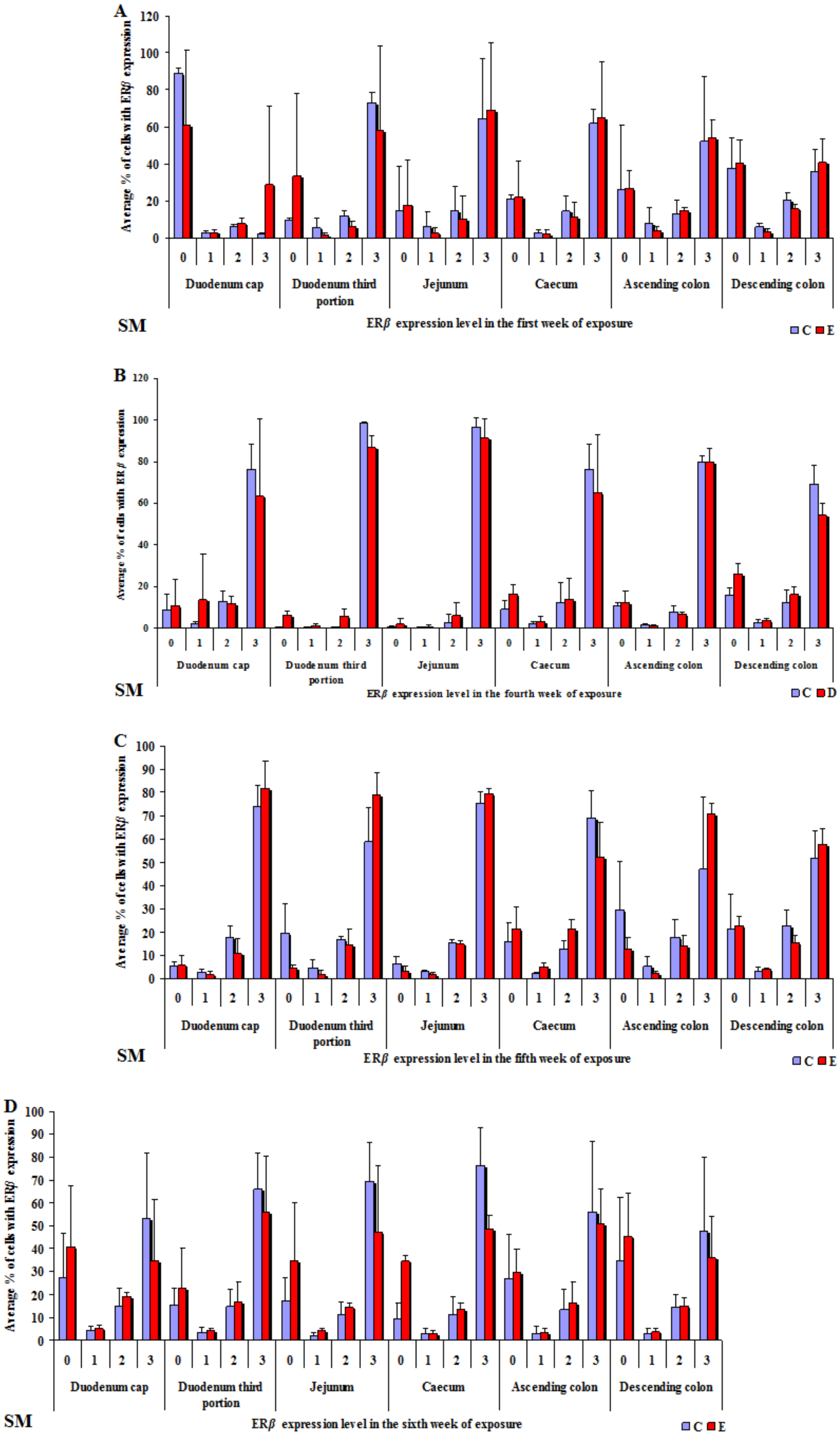

FIGURE SM 3 │ Immunohistochemical expression of ERβ (graded on a 4-point scale: negative = 0 points; weak and homogeneous = 1 point; mild or moderate and homogeneous = 2 points; intense or strong and homogeneous = 3 points) in different intestinal segments in selected weeks of exposure: A – in week I; B – in week IV; C – in week V; D – in week VI. ERβ expression was presented as mean values (±) and standard deviation (SD) for each sample. *P ≤ 0.05 were compared with the control group.

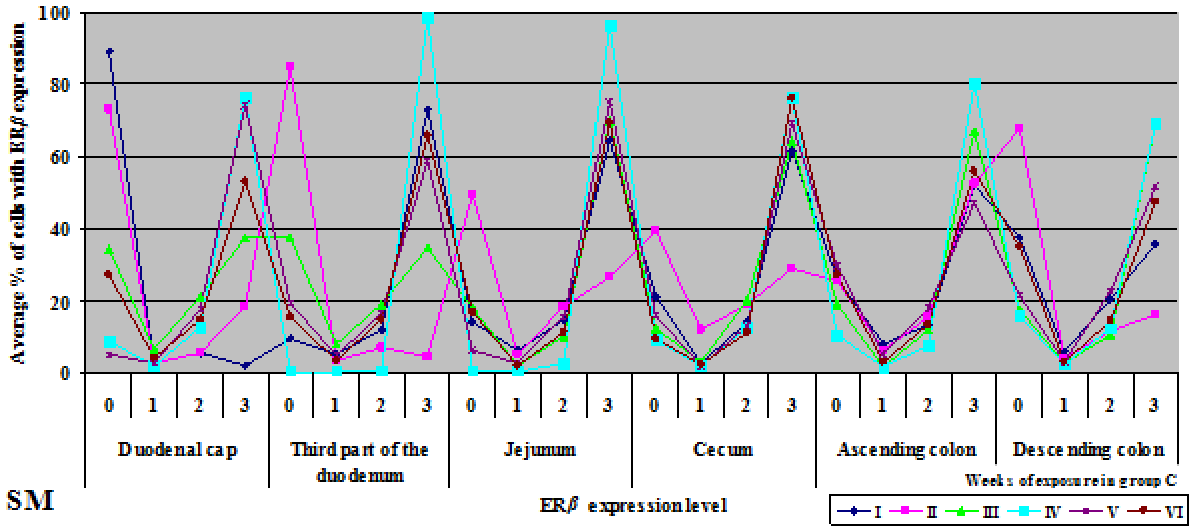

FIGURE SM 4 | Immunohistochemical expression of ERβ in the control group (graded on a 4-point scale: negative = 0 points; weak and homogeneous = 1 point; mild or moderate and homogeneous = 2 points; intense or strong and homogeneous = 3 points) in different intestinal segments and in selected weeks of exposure - average expression values from all weeks of exposure.

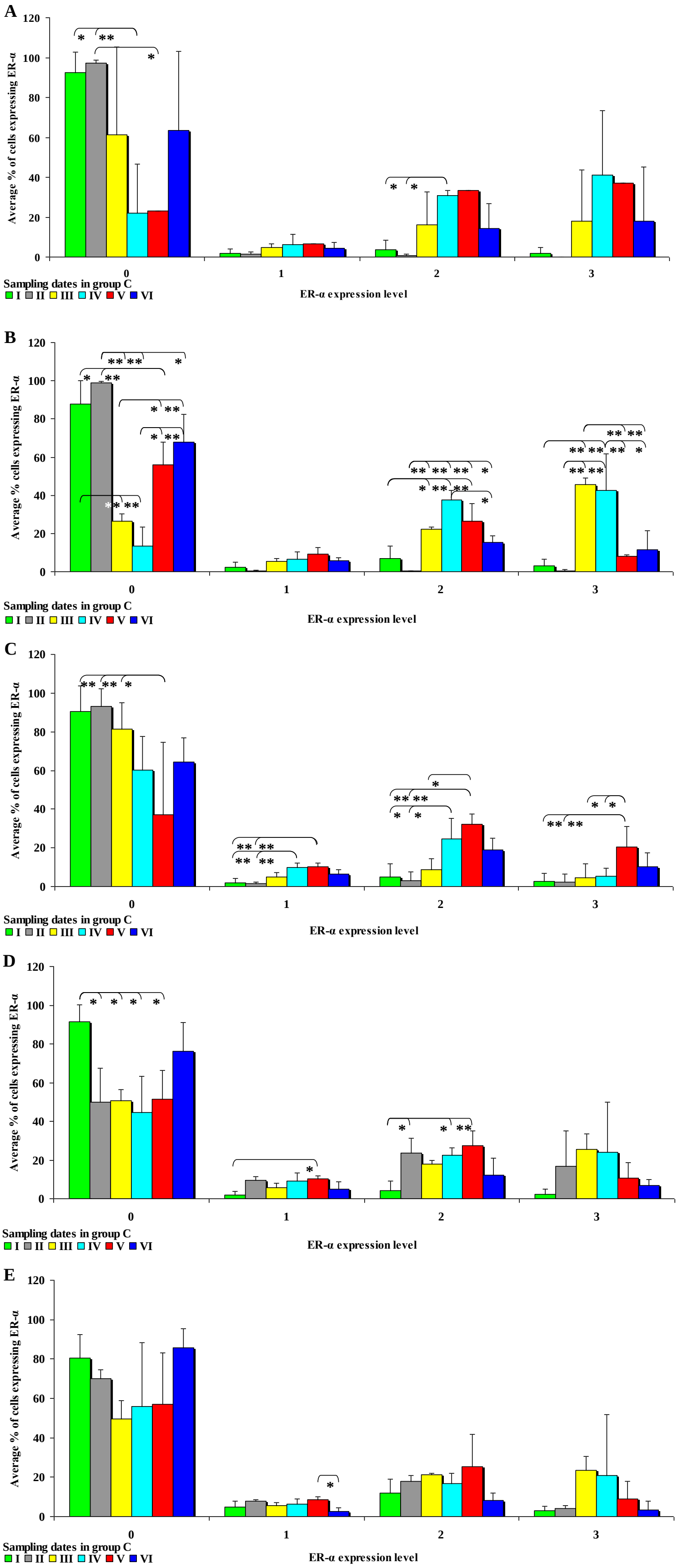

FIGURE SM 5 | Immunohistochemical expression of ERα (graded on a 4-point scale: negative = 0 points; weak and homogeneous = 1 point; mild or moderate and homogeneous = 2 points; intense or strong and homogeneous = 3 points) in the intestines of prepubertal gilts from group C: (A) in the duodenal cap in selected weeks of exposure; (B) in the third part of the duodenum in selected weeks of exposure; (C) in the jejunum in selected weeks of exposure; (D) in the cecum in selected weeks of exposure; (E) in the ascending colon in selected weeks of exposure. ERα expression is presented as mean values (±) and standard deviation (SD) in selected samples. *P ≤ 0.05 and **P ≤ 0.01 compared with the remaining groups.

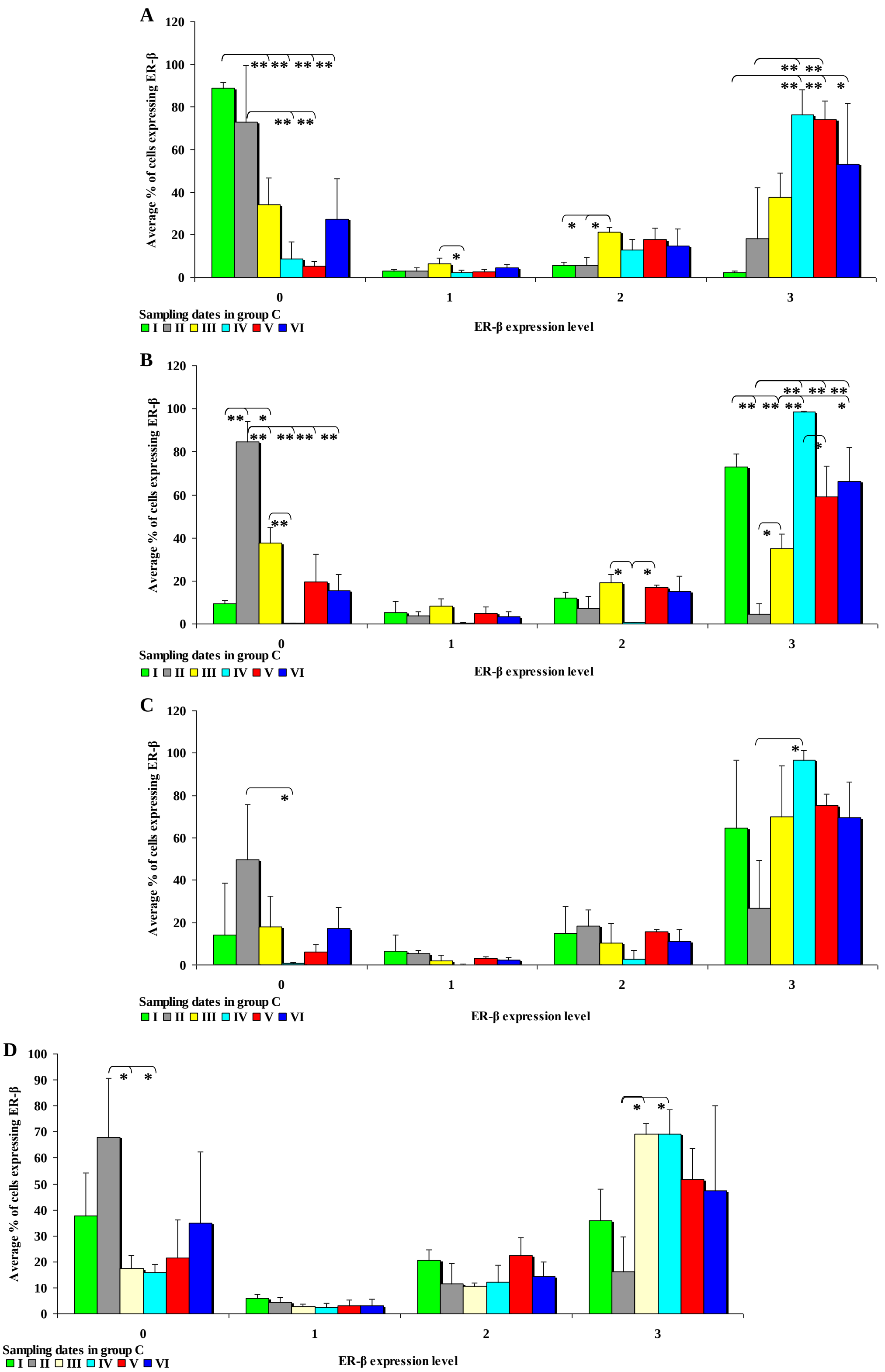

FIGURE SM 6 | Immunohistochemical expression of ERβ (graded on a 4-point scale: negative = 0 points; weak and homogeneous = 1 point; mild or moderate and homogeneous = 2 points; intense or strong and homogeneous = 3 points) in the intestines of prepubertal gilts from group C: (A) in the duodenal cap in selected weeks of exposure; (B) in the third part of the duodenum in selected weeks of exposure; (C) in the jejunum in selected weeks of exposure; (D) in the descending colon in selected weeks of exposure. ERβ expression is presented as mean values (±) and standard deviation (SD) in selected samples. *$P \leq 0.05$ and **$P \leq 0.01$ compared with the remaining groups.
